# Two novel mutations within *FREM1* gene in patients with bifid nose

**DOI:** 10.1186/s12887-023-04453-9

**Published:** 2023-12-14

**Authors:** Xiaoxue Chen, Baofu Yu, Zi Wang, Qingfeng Li, Chuanchang Dai, Jiao Wei

**Affiliations:** Department of Plastic and Reconstructive Surgery, Shanghai Ninth People’s Hospital Affiliated to Shanghai Jiaotong University School of Medicine, 639 Zhi Zao Ju Rd, Shanghai, 200011 People’s Republic of China

**Keywords:** Bifid nose, *FREM1* gene mutation, Tessier No.0, Frontonasal dysplasia

## Abstract

**Background:**

Bifid nose is a rare congenital deformity and the etiology is unknown. The purpose of this study was to report genetic variation in family of patients with bifid nose.

**Methods:**

Twenty-three consecutive patients who were diagnosed with mild bifid nose were operated with z-plasty from 2009 to 2021. Three underage patients (a pair of twins and a girl) from two family lines, who came to our hospital for surgical treatment, were enrolled. Whole exome sequencing and Sanger sequencing were conducted. Z-shaped flaps were created and the cartilago alaris major were re-stitched. Photographs and CT scan before and after surgery were obtained. Clinical outcomes, complications and patients’ satisfaction were evaluated and analyzed. The follow-up time ranges from 2 to 3 years (2.4 ± 1.2 years).

**Results:**

Most patients were satisfied with the outcome (96.2%). The nasal deformities were corrected successfully with z-plasty technique in one-stage. *FREM1* c.870_876del and c.2 T > C were detected with Whole exome sequencing, which have not been reported before. The results of Sanger sequencing were consistent with those of Whole exome sequencing.

**Conclusions:**

The newly detected mutations of *FREM1* have a certain heritability, and are helpful to make an accurate diagnosis and provide a better understanding of bifid nose mechanism. Z-plasty technique can be an effective technical approach for correcting mild bifid nose deformity.

**Supplementary Information:**

The online version contains supplementary material available at 10.1186/s12887-023-04453-9.

## Background

Bifid nose is a rare congenital anomaly with unclear underlying etiology. Patients with Tessier No.0 and 1 type craniofacial cleft usually presented obvious bifid nose [1]. Clinical presentations vary widely from a simple groove at the nasal tip to a maxillary cleft. Nonetheless, few cases have been reported because of its rarity. Neither optimum time for surgery nor universal agreement about a certain management has been established. Therefore, instructive diagnosis and treatment needs to be established.

The developmental origin of bifid nose has not been clearly clarified, as the vertebrate face development is remarkably intricate and dynamic [2]. Understanding early nasal development stages may aid in acquainting why certain phenotypes occur. There are three major tissue blocks in the mid and upper face: the frontonasal process (FNP), lateral nasal structures and the paired maxillary processes [2]. FNP fused with the maxillary primordia formats the midline tissue such as nasal bridge and nasal tip, which dates to the 4^th^ week of gestation. The paired maxillary processes form the upper jaw, cheek bones and lateral nasal structures. The FNP and maxillary processes are composed of migratory neural crest [3]. The growth and maturation procedure are orchestrated by complex tissue interactions, genes network and regulatory molecules. Bifid nose occurs when the midline two nasal processes are failure to fuse. Multiple signaling pathways such as FGF, Wnt, ZIC2, PAX3, BMP, TFAP2α, DLX5 and MSX1/2 regulate neural crest cells development [4]. It’s still unclear whether the malunion is caused by the alteration in the epithelial-mesenchymal interactions or other factors including chemicals, oligohydramnios, maternal metabolic imbalances, radiations and infection [1, 5].

The *FRAS1*-related extracellular matrix 1 (*FREM1*) is located at human chromosome 9p22.3 [6]. It is widely expressed in the developing embryo in regions of epithelial/mesenchymal interaction and epidermal remodeling, which can potentially affect the craniofacial and renal development [7]. *FREM1* protein belongs to the *FRAS1/FREM* family of extracellular matrix proteins which localizes at the basement membranes and forms a ternary complex including *FRAS1*, *FREM1* and *FREM2*. Recessive mutations in *FREM1* have been described to cause congenital diaphragmatic hernia and two rare syndromes—bifid nose with or without anorectal and renal anomalies syndrome (BNAR; OMIM #608,980) and Manitoba oculotrichoanal syndrome (MOTA; OMIM #248,450)—whose phenotypic characteristics overlap those seen in individuals with Fraser syndrome [8]. Therefore, the mutations in *FREM1* may correlate with bifid nose, and the specific mechanisms involved still need to be further studied.

In the present study, we detected new mutation sites of *FREM1* by whole exome sequencing and first-generation sequencing, which have not been reported in previous studies. The results can broaden the mutational spectrum of *FREM1* in bifid nose. Moreover, the use of simple Z-plasty surgery can be well used for correction of nasal deformity in patients with mild cleft nose. This surgical management was based on our experience of more than 10 years in the treatment of congenital craniomaxillofacial malformations.

## Methods

### Case 1 and 2

**Fig. 1:**
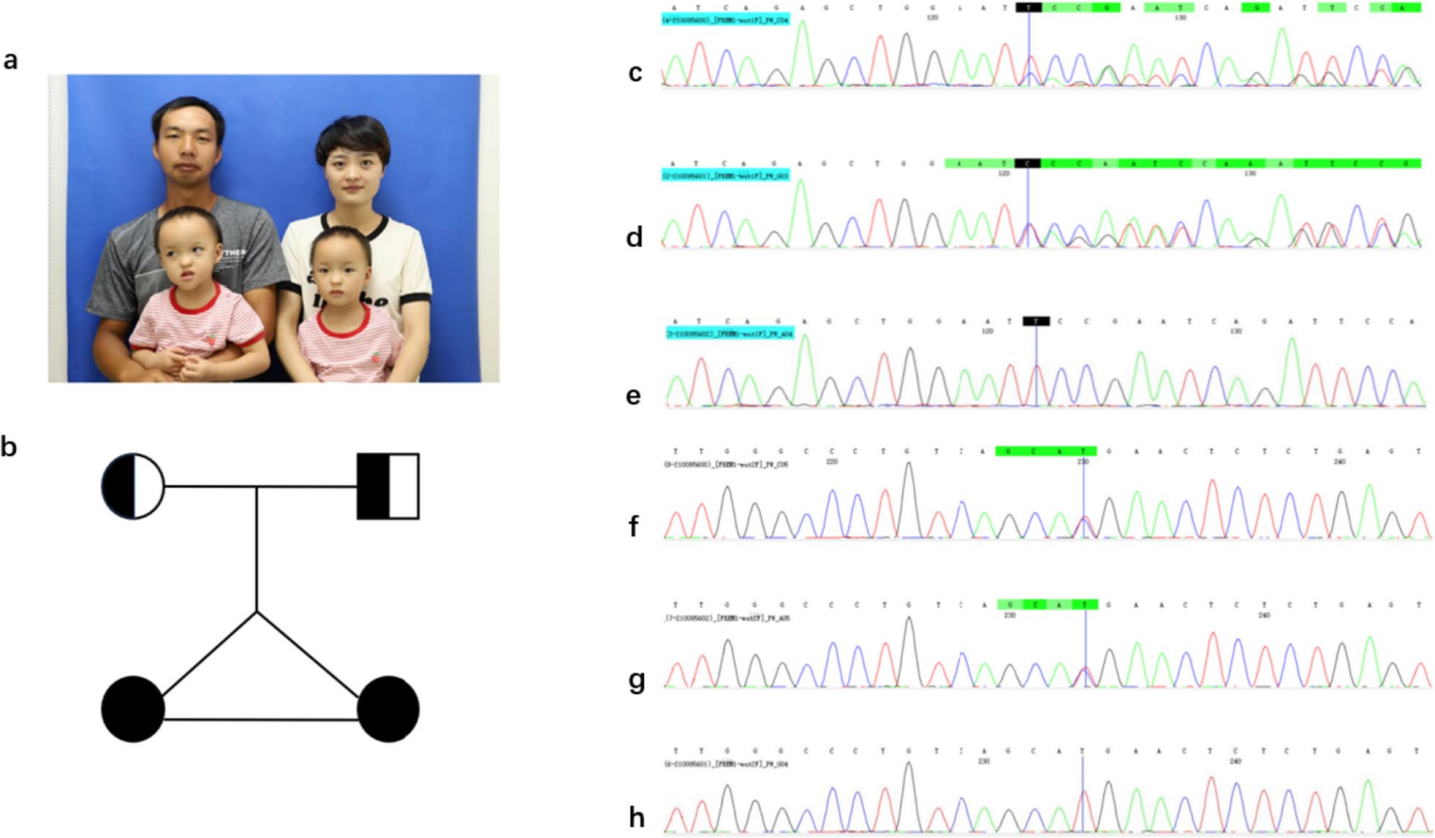
The twin sisters with bifid nose and their parents. **a** The photograph of them. **b** Genealogical tree. The heterozygotes mutation FREM1: NM_144966.7: exon7: c.870_876del: p. P291Rfs*20 was found in the twins (**c**) and their father (d), while their mother is normal (**e**). The heterozygous missense variation c.2 T > C was found in the twins (**f**) and their mother (**g**), while their father is normal (**h**)

**Fig. 2:**
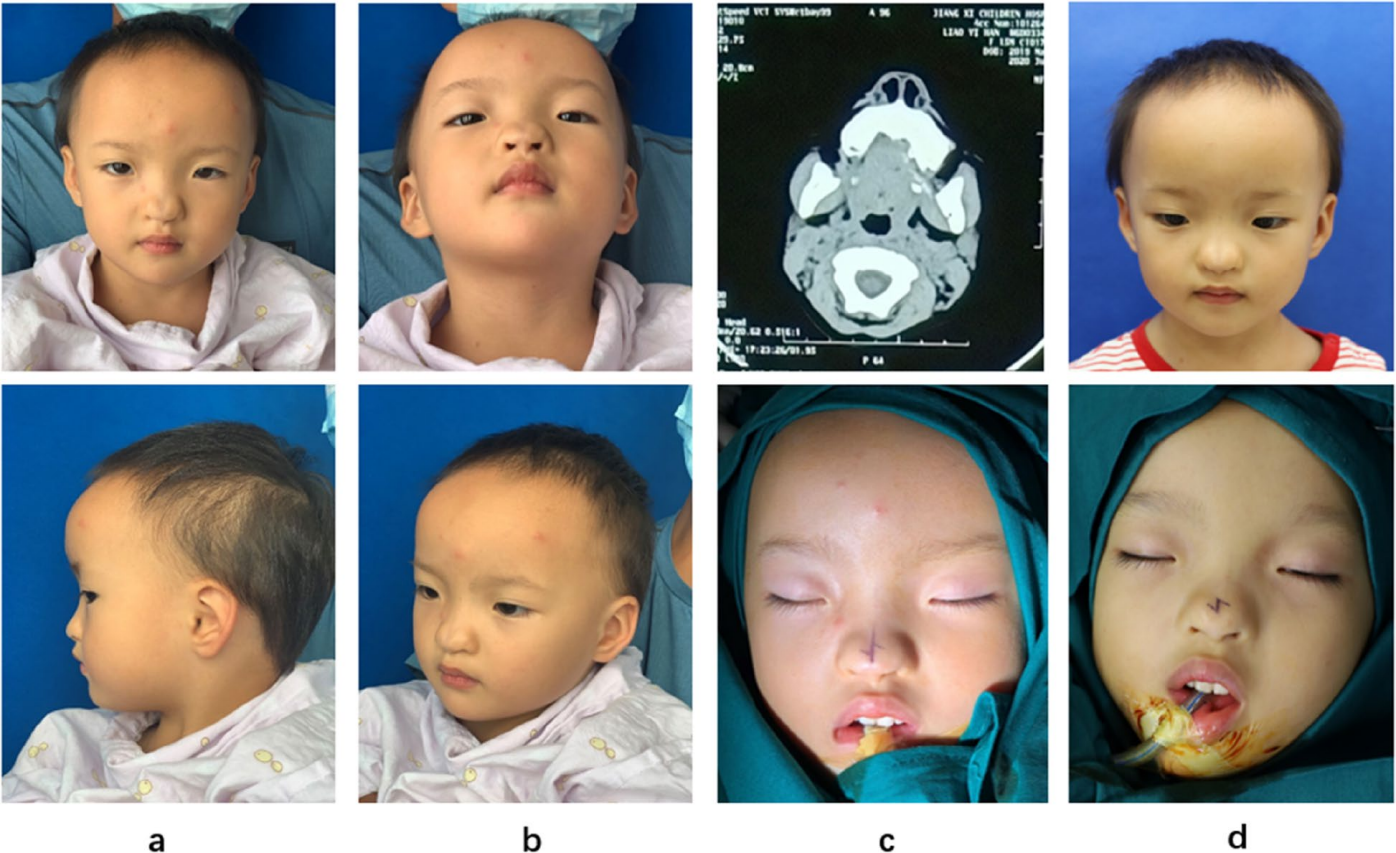
The twin sister photos before the surgery, CT scan with mild bifid nose and the intraoperative marking lines of the incision are shown. **a**-**c** The elder sister. **d** The younger sister

There is not any remarkable family history in the twin girls who were born with bifid nose (Fig. 1). The parents are not consanguineous. No fetal abnormalities were detected during antenatal care. Physical examination of the twins showed faintly grooved nasal tip, short and wide columella and separated alar cartilages. The preoperative computed tomographic scan also revealed bifid nose (Fig. 2). Clinical examination showed no cleft lip, ankyloglossia, hearing loss, gastrointestinal anomalies or ocular intelligence. There was also no skull defect, eyelid malformations, aberrant hairline, hypertelorism, ear defects or genitourinary anomalies. The cognition was normal. No other congenital anomalies were identified. There were also no associated malformations in the patients’ family. More radiology results are shown in Supplementary Fig. 1. The incisions are shown in Fig. 2. Patients’ photographs with frontal, lateral, and oblique views were taken before and after the surgery.

### Case 3

**Fig. 3:**
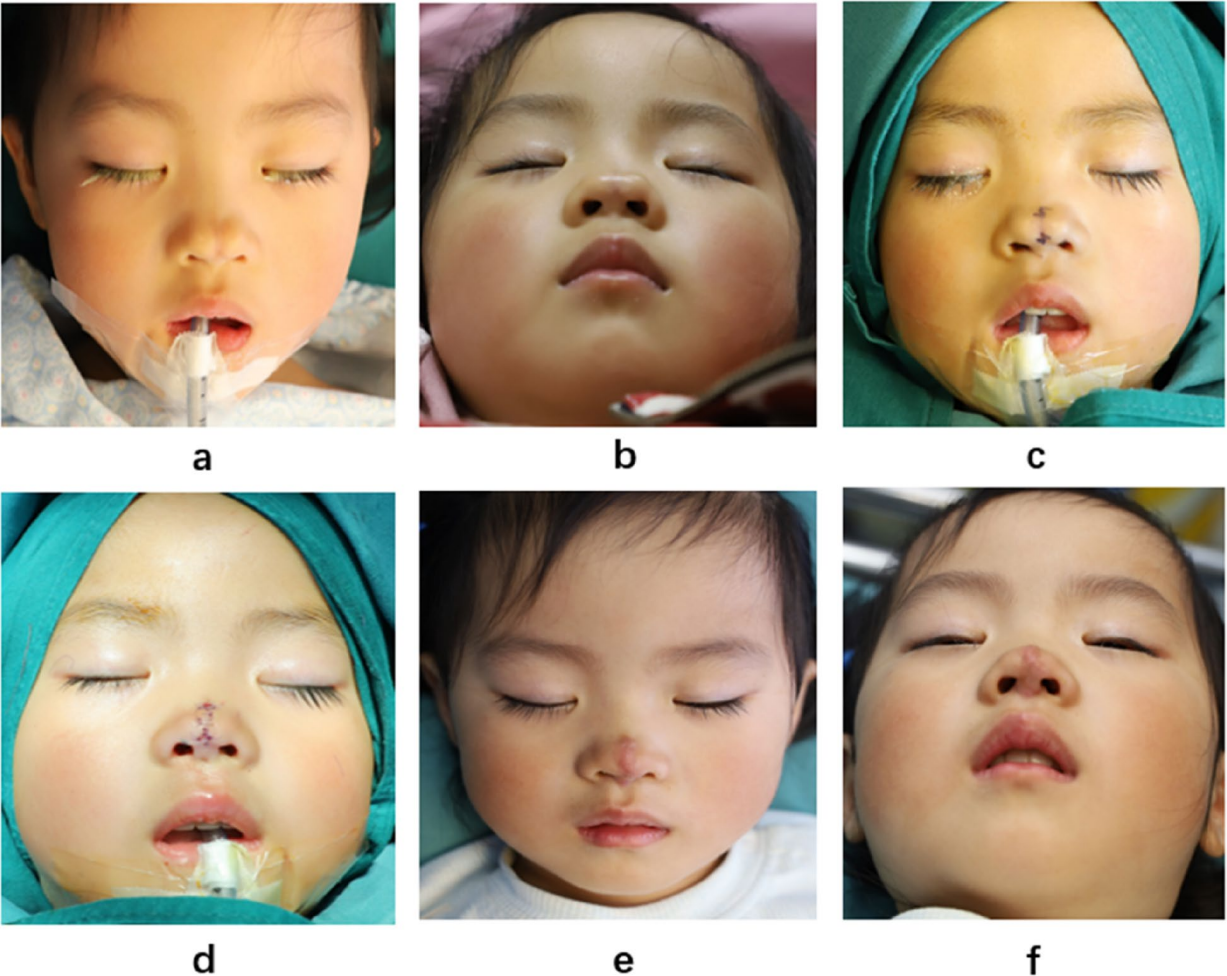
The 4-year-old girl with bifid nose. **a**, **b** Before surgery. **c** The surgical incision design. **d** Immediate postoperation. **e**, **f** A month after the surgery

A 4-year-old girl presented with bifid nose. She had a broad nasal dorsum, split nasal tip and less effective nasal tip protrusion (Fig. 3a, b). Clinical examination did not detect other abnormal symptoms. We designed two Z-plasty incisions and closed the cleft nasal tip (Fig. 3c).

Twenty-three consecutive patients with mild bifid nose were operated with this method from 2009 to 2021. Twenty of these patients declined to participate in the clinical report and had no other comorbid malformations. Thus, in the present study, we report a pair of twin sisters and a girl, who were featured by mildly depressed nose tip, bifid nose and mild nasal dorsum widening without other abnormal symptoms. To screen for mutations in the twins with mild bifid nose, whole exome and Sanger sequencing were performed on blood-extracted DNA from the patients and their parents. Quality control of the DNA was performed. Their DNA sequences were compared and analyzed with the published gene sequence. The Genome Analysis Toolkit (Broad Institute, Cambridge, MA, USA) was used to detect variants in the BAM file that passed quality control and a VCF format file was generated. Variants were annotated and filtered according to relevant clinical features of patient using Translational Genomics Expert platforms [9]. Suspected variants were confirmed by Sanger sequencing and validated using the parental test results.

Surgical reconstruction methods are also reviewed. All the patients in this series were treated following the same protocol. After written informed consent had been obtained from the legal guardian, bifid nose surgery using an open Z-shaped incision was performed. With the patients in supine position, all the surgical procedures were performed under general anesthesia. The operative incision line was outlined by methylene blue. After 1:200,000 epinephrine solution was locally injected for hemostasis, a Z-shaped incision was designed over the nasal dorsum according to the length and width of the patient's nose. The nasal dorsum skin and soft tissue were incised down to the dorsal fascial layer and septal cartilage. Bilateral nasal alar splits were observed during the operation, and bilateral cartilago alaris major were sutured with 5–0 PDS suture. Then intracutaneous and transcutaneous wound was closed with 5–0 absorbable suture and 6–0 PDS suture, respectively. The perichondrium is protected throughout the operation. We daily cleaned the scab and exudate from the wound by normal saline within 1 week after operation, and then the stitches were removed and silicone scar-removing medicine was applied.

Follow-up was performed once a month within 3 months after surgery, and once in every 3–6 months after three months. All patients’ specimens, CT and photographs were obtained at 1 and 6 months after operation. The aesthetic outcomes, scar and continuity of nose tip curvature were evaluated basing on patient’s satisfaction (Table 1).

**Table 1:** Postoperative patient saisfaction survey

**Degree of satisfaction**		**Evaluation standards**
Very satisfactory	Aesthetical outcome	Significant correction of nasal subunits
	Incision scar	Linear incision scar inconspicuous in color and texture
	Continuity of nose tip curvature	Good continuity
Mostly satisfactory	Aesthetical outcome	Minor imperfections of nasal subunits
	Incision scar	Minor scar formation and discomfort
	Continuity of nose tip curvature	Continuity is acceptable
Unsatisfactory	Aesthetical outcome	No obvious improvement of nasal deformity
	Incision scar	Hypertrophic scar and discomfort
	Continuity of nose tip curvature	Discontinuous

## Results

The follow-up time ranges from 2 to 3 years (2.4 ± 1.2 years), and there are 23 patients in total. The age of them ranges from 2 to 12 years old (average 4.8 years old). During the early postoperative stage, there was mild ruddy incision during the first three months. One patient had a mild infection after surgery, which was treated by partial iodophor disinfection and oral antibiotics for 3 days. Healing procedure was uneventful. There were no complications of flap ischemia, necrosis and poor healing. The wound healed primarily within about 2 weeks and the scars became invisible 3–6 months after surgery. The parents of patients were much satisfied with the aesthetical outcome (94.3%), incision scar (97.1%) and continuity of nose tip curvature (97.1%). The average very and mostly satisfactory percentage is 96.2% (Table 2).

**Table 2:** Analysis of patient satisfaction

	**Aesthetical Outcome**	**Incision scar**	**Continuity of nose tip curvature**	**Percentage (mean)**
Very satisfactory (no. patients)	21	20	20	88.4%
Mostly satisfactory (no. patients)	1	2	1	5.8%
Unsatisfactory (no. patients)	1	1	2	5.8%
Very and mostly satisfactory	94.3%	97.1%	97.1%	96.2%

### Case 1 and 2

**Fig. 4:**
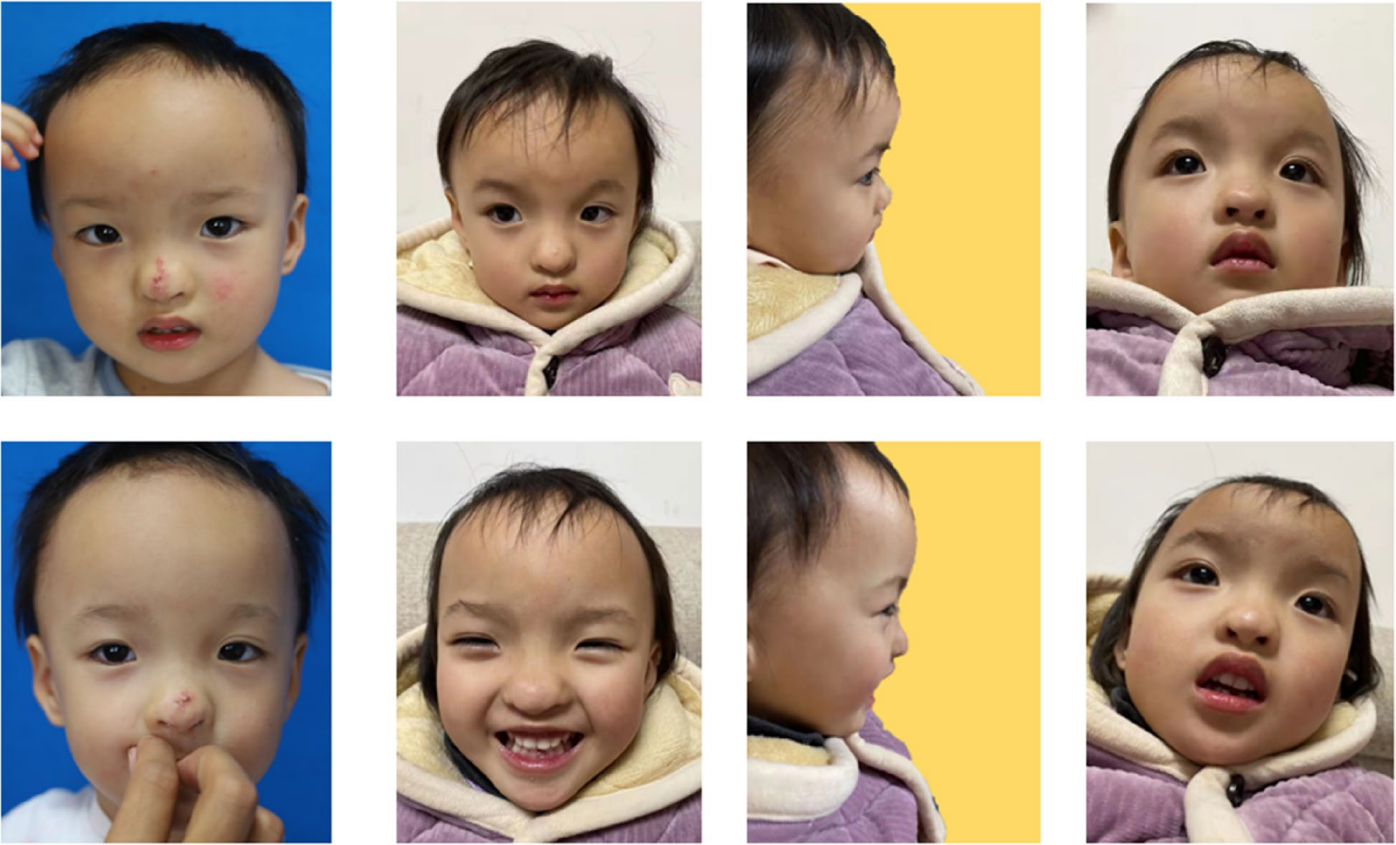
Immediate postoperation (1st column) and eight months (2nd, 3rd and 4th column) after surgery. The upper: the elder sister. The lower: the younger sister

The postoperative photographs of the twins are shown in Fig. 4. During the postoperative early stage, no skin necrosis was observed and vascular perfusion was good. Parents were satisfied with the results.

### Case 3

Postoperative photographs are shown in Fig. 3d-f. The nasal deformity was corrected via one-stage operation and the patient and her parents were satisfied.

### Genetics

The case 3 girl's family refused genetic sequencing, and they only agreed to report the girl’s photos and clinical information for academic exchange; thus, we only performed genetic test on the twins. Two novel mutations in the *FREM1* gene were found in the twins: heterozygous frameshift mutation c.870_876del:p.P2 91Rfs*20 and heterozygous missense variation c.2 T > C:p.M1?**.** Both gene variants have not been previously reported. The former is not listed in public databases (gnomAD) and the East Asian general population frequency is 0 in the gnomAD database for the latter. The former mutation was also found in the father with normal phenotype and the latter was found in the mother with normal phenotype (Fig. 1; Table 3).

**Table 3:** Two novel mutations in the FREM1 gene were found in the twins

**Gene**	**hg19 location**	**RS number**	**Variant naming**	**gnomAD_ EAS crowd frequency**	**Zygote type**	Kin zygote type	
						**Father**	**Mother**
FREM1	chr9:14851562_14851568	/	FREM1:NM_144966.7:exon7:c.870_876del:p.P2 91Rfs*20	/	Heterozygotes	Heterozygotes	Wild type
FREM1	chr9:14,868,974	rs1464587064	FREM1:NM_144966.7:exon3:c.2 T > C:p.M1?	0	Heterozygotes	Wild type	Heterozygotes

## Discussion

Bifid nose, also referred to as a double or cleft nose, manifests in a diverse array of phenotypes and clinical entities. It results from abnormal embryological development of the nose. In 1976, Tessier observed more than 300 cases basing on his experiences and established the craniofacial clefts classification. The clefts were classified into 0 to 14 types according to their relationship to the zero line [10]. Middle bifid nose was commonly classified as Tessier No.0 craniofacial cleft [1]. Most cases are sporadic. Clinical presentations are complex and appear different degrees of severity. The nasal septum can be duplicated, thick or absent, and alar cartilages can be separated and nasal tip may be faintly or deeply furrowed [1]. Many other anomalies can be associated with bifid nose, such as cleft lip, orbital hypertelorism and even deformity of other systems like genitourinary [2].

Genomic technology advent has aided in profound change in many aspects, especially for rare genetic disorders. Bifid nose often overlaps with other complex syndromes, and molecular testing is critical. Gene identification promotes molecular diagnosis and gene identification. Multiple genetic mutations have been reported to be associated with bifid nose. Anyane-Yeboa et al. reported five bifid nose individuals of a family and proposed it was likely an autosomal dominant trait in 1984 [11]. Toriello et al. made a similar point [12]. With the advancement of detection technology and the reporting of more cases, many genetic mutations have been reported to be associated with the occurrence of bifid nose. Pai syndrome can represent bifid nose and a de novo apparently balanced reciprocal translocation, 46,X,t(X;16) was described [13]. Gene mutation in *EFNB1* can result in bifid nose, such as c.373G > A [14], c.270_271del [15] and c.451G > A [16]. *ZIC2*(c.1599 * 954 T > A) [17]、*PORCN*(c.727C > T) [18]、*TBX1*(c.1132G > A) [19] have also been reported to present bifid nose. Frontonasal dysplasia resulted from *ALX1*, *ALX3*, *ALX4* can also present bifid nose [2]. There have been many case reports of MOTA syndrome and BNAR syndrome, which are also related to the *FREM1* gene. The patients with MOTA syndrome may present a broad or bifid nasal tip, cryptophthalmos, microphthalmia, eyelid colobomas, an aberrant hairline, and gastrointestinal anomalies such as omphalocele and anal stenosis [6].

Herein we report two novel mutations in *FREM1* gene: heterozygous frameshift mutation c.870_876del and heterozygous missense variation c.2 T > C. They have not been reported previously. The novel frameshift variant c.870_876del causes premature termination codon and variant c.2 T > C causes the start lost. As a quality control pathway, nonsense-mediated decay may remove the premature termination codons, which is a possible alternative pathogenic mechanism [20]. Only a dozen different *FREM1* mutations have been reported, and few animal models have been described [21]. *FREM1* protein concludes 12 chondroitin sulfate proteoglycan (CSPG) repeats, a putative signal sequence, a calx-β domain and a C-terminal type C lectin-like domain [7]. *FREM1* is widely expressed in some neural crest mesenchyme, it can be found in many syndromes such as Bifid Nose Renal Agenesis and Anorectal malformations (BNAR) and Manitoba-oculo-tricho-anal (MOTA) [22]. The interaction between different cell types and the availability of different extracellular ligands for the cognate receptors are thought to have the participation of *FREM1* [23]. *FRAS1*, *FREM1* and *FREM2* gene have been shown to encode a group of extracellular matrix proteins, forming a ternary complex which locates at the basement membrane [24]. Therefore, the correct expression of *FREM1* is necessary for the normal development of nasal morphology. The interactions will be disrupted if there are loss-of-function *FREM1* mutations. There have been several reported variations, such as loss of the exons 19 to 30 [7], exon 8–23 deletion [6], heterozygous c.3939 A > C (p.Y1313X) variant at exon 23 and heterozygous c.580G > A (p.R194X) variant at exon5 [25]. The animal models of related research have also been mature [6, 24, 26–28]. However, the relationship between the specific mutation site and the phenotype has not established, which needs continuing to be explored. Chacon-Camacho et al. [21] summarized 27 patients with FREM1 mutations, we sorted other reported patients in Table 4. We found that the patients with mutations in FREM1 generally had changes in nasal morphology, but the symptoms in other areas vary. Gender is also an influencing factor [7], we hypothesize that this is why this pair of twins has bifid nose and no other symptoms. This article further supplements the understanding of bifid nose-related genes.

**Table 4:** Reported FREM1 gene alterations and corresponding clinical presentation

**References**	**Genetic changes**	**Craniofacial findings**	**Nasal findings**	**Limb findings**	**Genitalia findings**	**GIT findings**	**Development findings**	**GUT findings**	**Hernia**	**Other findings**
[29]	c.4629delC + c.3971 T > G	bilateral incomplete cryptophthalmos with keratinized cornea, hypertelorism, large mouth with a small upper lip, circle-shaped whirl of hair on the left forehead, growing into the eyebrow, dysplastic ears	wide nasal bridge and tip of the nose	cutaneous syndactyly 3/4 of both hands, syndactyly 2/3 of the right foot	-	-	-	mild pyelectasis on the left kidney	\	\
[8]	∼86 kb deletion^a^ + c.5334 + 1G > A	\	-	mild 2–3 toe syndactyly	-	-	-	-	diaphragmatic hernia	\
[30]	c.2721delG	\	bifid nose	-	-	anorectal malformation	\	renal agenesis	-	\
	c.1945C > T	\	-	-	-	-	\	renal agenesis	-	\
	c.4318G > A	\	bifid nose	-	-	-	\	renal agenesis	\	airway malformation
[22]	c.2148G > T + c.3820G > T	eyelid coloboma, hypertelorism	bifid nasal tip	\	\	anterior anus, anal stenosis	\	renal pelviectasis, vaginal atresia	\	\
	c.6139delG^Homozygous^	corneopalpebral synechiae, eyelid coloboma, aberrant hairline, hypertelorism	bifid nasal tip	\	\	-	\	\	\	\
	c.6139delG	-	bifid nasal tip	\	\	-	-	\	\	\
	c.5648C > G	corneopalpebral synechiae, eyelid coloboma, aberrant hairline, hypertelorism	bifid nasal tip	\	\	-	mild delays	renal agenesis	\	\
[25]	c.3939 A > C + c.580G > A	aberrant unilateral wedge-shaped anterior hairline with the loss of ipsilateral eyebrow, hypertelorism, ipsilateral medial eyelid colobomas	bifid nose	\	\	-	\	\	\	\
[7]	homozygous 9p22.3 microdeletion in the siblings, size 30 to 52 kb encompassing several exons of FREM1	brachycephaly, bushy eyebrows, low-set posteriorly rotated and overfolded ears, teeth anomalies (diastasis of the central incisors, ab- size and shape of teeth and ab-ity of dental enamel) and short oral frenula	bifid nose	\	precocious puberty	-	mild intellectual disability	unilateral renal agenesis	\	congenital heart disease
	homozygous 9p22.3 microdeletion in the siblings, size 30 to 52 kb encompassing several exons of FREM1 (NM_144966)	-	bifid nose	\	-	\	-	-	\	\
[31]	c. (? _ 1); (1393 + 1_1394-1)del	isolated midline craniosynostosis		\	\	\	\	\	\	\
[32]	c.4023C > G + c.4564G > A + c.4789G > T	craniosynostosis	\	\	\	\	\	\	\	\
	c.916_936dup	craniosynostosis, papilledema	\	\	\	\	\	\	\	\
[33]	c.4705C > T	aberrant hairline, lack of eyebrows, widely-spaced eyes (42 mm), bilateral palpebral coloboma	bifid nasal tip	\	\	\	neonatal hypotrophy, generalized hypotonia, short stature	unilateral renal agenesis	\	\
[34]	c.1157A > C + c.5057C > T	hydrocephalus	-	short limbs	-	\	\	-	\	\
[27]	Del ex10-37	trigonocephaly, midface hypoplasia	short/flat	\	-	\	delay	-	\	\
	Dup ex1-6; Del ex7-37	trigonocephaly, midface hypoplasia	short/flat	\	-	\	delay	-	\	\
	Del	trigonocephaly, midface hypoplasia	short/flat	\	cryptorchidism	\	delay	-	inguinal hernia	pulmonary stenosis/incompetence
	Del	trigonocephaly, midface hypoplasia	short	\	-	\	delay	-	inguinal hernia	peripheral pulmonary stenosis
	Del	trigonocephaly, midface hypoplasia	short	\	-	\	delay	-	\	ventriculo-septal defect
	c.4499A > T	trigonocephaly	\	\	\	\	delay	\	\	cardiac malformations
	c.4499A > T	trigonocephaly, midface hypoplasia	broad bridge	\	-	\	-	mild right pelvicaliceal dilatation	\	\
	c.1493G > A	trigonocephaly	flat nasal bridge	\	-	\	-	-	\	\

^a^minimal deleted region chr9:14,892,957–14,941,672, maximal deleted region chr9:14 869 861–14 955 988; hg19. -, normal. \, not available

As the bifid nose is the most common craniofacial cleft, many surgical techniques have been proposed basing on personal experience and preference. However, no surgical technique has been universally accepted. The surgical treatment still present great challenge due to limited number of publications and complexity of malformation. Nasal deformities correction concludes skeletal and soft tissue malformation. ROE first proposed public correction of bifid nose in 1887, and a second stage completion surgery was first advised by Kazanjian and Holmes [1]. Kurokawa performed dermal graft via the nasal dorsum and applied on the nasal apex [35]. Ali Tawfik combined Millard forked flap with external rhinoplasty and successfully helped six patients. It increased the scar and secondary operation was usually needed [1]. Tuersunjiang et al. made an inverted-V transcolumellar incision, modified the shape of nose and achieved good results [36]. Rib cartilage has inherent structural advantage. Many surgeons recommend rib cartilage as the best autologous material in rhinoplasty. In 1917, Selfridge first emphasized rib cartilage as nasal reconstruction graft material [37]. Recently more and more nasal reconstruction via rib cartilage have been reported [38]. Researchers used autologous bone tissue or cartilage to treat deformity and got good functional and aesthetic outcomes [39, 40]. However, some surgeons avoid using cartilage [41]. First, 2 anatomic sites prolong the time of operation and staying in the hospital, which not only results in a late discharge but also increases the hospital expenses. Some patients can’t afford it. Second, chest tube insertion and pneumothorax might occur during operation. The occurrence of these unexpected situations can bring other troubles. Third, the cartilage may twist or bend postoperatively. If so then the secondary operation is needed and patients will express dissatisfaction. Fourth, cartilage may occur calcification. Notably, this method is not suitable for children because they are growing and developing. The operation will affect their cartilage development. What’s more, these patients with mild bifid nose don’t need to raise their nose. Overall, diverse surgical methods have been proposed but they each have their pros and cons. There is still no widely accepted approach. Although there have been some studies to use local flaps, surgical treatment for mild bifid nose is rarely reported. We aim to identify a technique to achieve more permanent and effective correction of mild bifid nose. The Z-shaped incision could be an ideal option to help patients who just have a small groove at the tip of nose. The results were stable and pleasant. Most importantly, the skin is much coherent in this method and excess skin doesn’t need to be excised. The excess skin facilitates later implantation of cartilage or prosthesis when the child is older. Otherwise the later improvement is limited. This technique described in this article can be applied to all the similar patients. We have improved the aesthetics as much as possible with the smallest scar.

The optimum age of plastic surgery for bifid nose is an arguable issue. Some surgeons recommend not to operate on the pediatric nose because there is potential damage to the nasal growth. Doval et al. [32] retrospectively found that surgeries performed on child still have a good effect. We prefer the patients being operated at the age of between 3 and 6 years old. The anesthesia of children who are too small may affect the nervous system of children, and children will have a stronger self-awareness of appearance after the age of 6. Otherwise the patients may suffer teasing while dealing with their mates.

The surgical treatment proposed in this article can address problems such as chromatic aberration and low survival rate, which often occur in local skin flaps. The procedure is simple but can effectively improve the patients’ appearance. No serious surgical complications have been found so far except mild infection or edema. The limitations of this approach include the number of patients is relatively small and the follow-up time needs extension. The genetic test included only one set of twins and their parents, and samples from more patients could provide richer results. Most importantly, the surgical method presented is only suitable for simple short and wide nose tip, it is not suitable for more serious ones.

Our discoveries enrich the understanding of bifid nose and broaden the mutational spectrum, which enables more patients to receive personalized treatments. It’s still needed to be open to unexpected scenarios such as richer genetics understanding and better surgical methods, in the continuing way for the bifid nose.

## Conclusions

In this study, we detected two new mutations in the related gene-*FREM1* in patients with nasal clefts, which have not been reported in previous studies. The use of simple Z-plasty surgery can correct mild nasal deformity via one-stage operation. This study can provide some reference for the study of genetic related factors of nasal cleft and the surgical strategy of patients with mild nasal cleft.

### Supplementary Information

**Additional file 1:** **Supplementary Fig 1.** Chest and cranial CT images of the twins. All indicators are normal.

## Data Availability

The datasets analysed during the current study are available in the clinVAR repository. https://submit.ncbi.nlm.nih.gov/subs/variation_clinvar/SUB13938237/ (Submission ID: SUB13938237), https://submit.ncbi.nlm.nih.gov/subs/variation_clinvar/SUB13938365/ (Submission ID: SUB13938365).

## Abbreviations

FREM1: FRAS1-related extracellular matrix 1
FNP: Frontonasal process
CSPG: Chondroitin sulfate proteoglycan
BNAR: Bifid Nose Renal Agenesis and Anorectal malformations
MOTA: Manitoba-oculo-tricho-anal
