# Supplementary figures and images for: Two novel mutations within FREM1 gene in patients with bifid nose
Source: BMC Pediatr. 2023 Dec 14;23:631. doi: 10.1186/s12887-023-04453-9 (PMC10720098; doi:10.1186/s12887-023-04453-9)

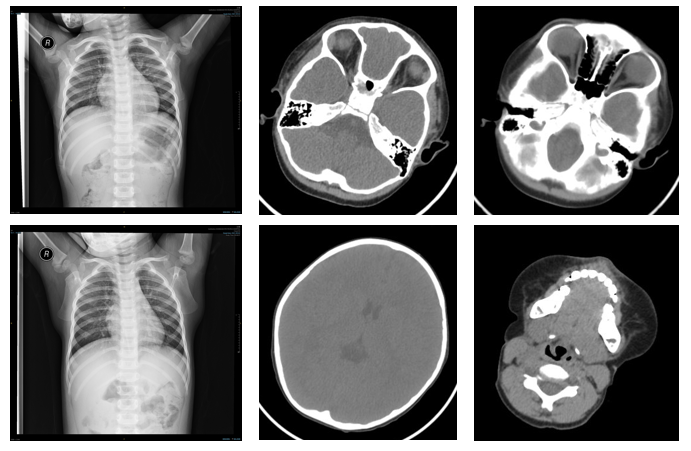


**Supplementary Fig 1. Chest and cranial CT images of the twins.** All indicators are normal.

Supplement: Supplementary file 1 — Additional file 1: Supplementary Fig 1. Chest and cranial CT images of the twins. All indicators are normal. [file 12887_2023_4453_MOESM1_ESM.docx]
